# Ventricular repolarization indicators in risk stratification of decompensated heart failure patients with ventricular systolic dysfunction

**DOI:** 10.22088/cjim.13.3.533

**Published:** 2022

**Authors:** Mohammad Reza Hatamnejad, Hamed Bazrafshan, Morteza Hosseinpour, Peyman Izadpanah, Mohammad Reza Kasravi, Mehdi Bazrafshan

**Affiliations:** 1 Faculty of Medicine, Shiraz University of Medical Sciences, Shiraz, Iran; 2 Department of Cardiology, Shiraz University of Medical Sciences, Shiraz, Iran; 3 Faculty of Medicine, Shahid Beheshti University of Medical Sciences, Tehran, Iran; 4 Al-Zahra Charity Hospital, Department of Cardiology Medicine, Shiraz University of Medical Sciences, Shiraz, Iran

**Keywords:** QT dispersion, QTc interval, Decompensated heart failure, Ventricular systolic dysfunction

## Abstract

**Background:**

Ventricular repolarization measurement by QTc interval and QT dispersion can recognize high-risk patients. Previous research tended to evaluate the act of repolarization indicators alone but this study aimed to elucidate their prognostic utility before and after modifying confounding parameters in risk stratification of different aspects of prognosis in decompensated heart failure patients with systolic dysfunction.

**Methods:**

Data of 98 variables were evaluated to determine their predictive value concerning arrhythmic events, in-hospital, and long-term mortality.

**Results:**

From 858 cases that presented with acute heart failure, 19.2% (n=165) were enrolled in the study. During hospitalization, arrhythmic events and cardiac-related mortality occurred in 56(33.9%) and 11(7%) patients, respectively. QTc and QT dispersion were independent predictors of arrhythmia and in-hospital mortality after adjustment of the variables (arrhythmic events: QTc interval OR 1.085, P=0.007, QT dispersion OR 1.077, P=0.007, in-hospital mortality: QTc interval OR 1.116, P=0.009, QT dispersion OR 1.067, P=0.011). After being discharged, they were tracked for 181±56 days. Within the 16 deaths in follow-up time, 6 sudden cardiac deaths were documented. Cox regression, defined QTc as the predictor of all-cause and sudden death mortality (all-cause: HR 1.041, 95% CI 1.015-1.067, P=0.002; sudden death: HR 1.063, 95% CI 1.023-1.105, P=0.002); nevertheless, efforts to demonstrate QT dispersion as the predictor failed.

**Conclusion:**

The predictive nature of QT parameters was significant after modification of the variables; therefore, they should be measured for risk stratification of ventricular repolarization arrhythmia and death in decompensated heart failure patients.

Gradual or abrupt demonstration of heart failure breakdown signs and side effects that leads to unplanned meeting in the office or emergency ward can be described as acute decompensated heart failure (DHF) (1). The concomitant rising in DHF hospitalization has occurred with the increased prevalence of chronic heart failure. In addition to hospitalization costs, extraordinarily high amounts of morbidity and mortality related to DHF lead to an enormous financial burden on the health network (2). Rapid intervention in high-risk patients contributed to decreasing mortality, rehospitalization, and eventually lower medical expenditure. Ventricular repolarization measurement by QTc interval and QT dispersion (QTd) can distinguish high-risk patients for arrhythmia and mortality (3).

Although the literature has addressed QTc interval or QTd as a predictor of arrhythmia and mortality in acute decompensated or chronic heart failure (4-11), less has been done to elucidate their prognostic role after adjustment for a variety of confounding factors in a model. According to this aim, we did the study to illuminate their prognostic utility before and after modifying confounding parameters in risk stratification of different aspects of outcomes including (1) arrhythmia and mortality during hospitalization, and (2) long-term mortality in DHF patients with ventricular systolic dysfunction.

## Methods

Our work with the prospective design was conducted at Al-Zahra Charity Hospital, a university-affiliated tertiary medical center in Shiraz, from December 2019 to April 2020. The investigation convention has been endorsed by the college ethics committee (ethical code: IR.SUMS.MED.REC.1399.248); the Declaration of Helsinki was preserved in all the study phases. Informed assent was obtained before the participation.

**Study population: **Inclusion criteria for enrollment of the patients presenting with decompensated heart failure symptoms (including dyspnea, fatigue, decreased exercise or physical capacity, anorexia, early satiety, weight loss, weight gain, palpitations, peripheral edema, ascites, and disordered breathing according to ACCF/AHA Guideline (12)), in the study were: age more than 18-years, previously known case of heart failure, class III-IV of New York Heart Association (NYHA), and attendance of systolic dysfunction (ejection fraction (EF) below the 40%). The patient will be excluded on condition of having any final diagnosis except DHF; lack of sinus rhythm in electrocardiogram (ECG); low quality of ECG to interpret; pacemaker, bundle branch block, and other causes of widened QRS complex; other causes related to prolonged QT parameters such as electrolyte imbalance, consuming the drugs that are associated with prolonged QT; recent myocardial infarction; dissatisfaction with participation in the study.

**Clinical Variables: **Information on demographic, clinical, and medical variables was obtained from the emergency ward. Previous medical documents and any other archived data were reviewed to specify the etiology of heart failure, and the initial ECG at the emergency room was recorded for subsequent analysis. A blood sample was sent for laboratory tests of complete blood count, hemostasis tests, cardiac biomarkers, lipid profile, electrolytes, renal and hepatic function. Radiologic studies (chest radiography or high-resolution computed tomography) were applied if necessary. Echocardiography to verify the presence of systolic dysfunction concerning the EF value was done. During hospitalization cardiac monitoring, subsequent ECGs were recorded to identify premature ventricular contraction, ventricular tachycardia, and ventricular fibrillation. During the decompensated stage, the standard treatment was given. A telephone call was made at the end of every 90 days after the patient’s discharge to get to know about the clinical condition, hospital readmission, and death.

**ECG analysis: **A prospective analysis of the ECG recorded at the first visit to the emergency room was performed to determine the computer-derived heartbeats, QRS duration, QT, and QTc interval. Heart monitoring and subsequent ECGs were analyzed for the arrhythmic event. ECGs’ data were digitally recorded for 8 seconds with a pace of 25 mm/sec and an amplitude of 10mm/mv (cardiax system 4.25.5). Inspecting the ECGs to manually compute the QTd, confirming the accuracy of the computer-based QT parameters by digital caliper, and excluding the patients with bundle branch block from the survey was accomplished. The QT interval is started with the QRS complex and is ended at the T-wave termination (whenever it returns to the isoelectric line). In the presence of the U-wave, end of the QT interval is defined as a point in the nadir of the curve between the T and U waves. Calculating the mean of 3 uninterrupted beats in at least 6 leads to obtain the QT interval, wherever possible, was taken. Correcting QT interval (QTc calculation) was done via Bazett’s formula except for individuals with tachycardia that Hodges formula was more appropriate (13). The gap between the highest and lowest value of QT interval is described as QTd. Prolonged QTc interval and QTd have been defined as more than 440 ms and 80ms, respectively (14). Patients were tracked for 181 ± 56 days. Information on endpoints was gathered by telephone call at the end of every 90 days. No cases were dropped during the follow-up.

**End Points: **The initial endpoints were arrhythmic events during the hospital course and all-cause mortality either in-hospital or long-term. Arrhythmic events were described by the presence of premature ventricular contraction, ventricular tachycardia, and ventricular fibrillation. The status of the DHF patient shifts to chronic stable heart failure with the proper management and after the discharge. Thus, the type of mortality at the time of the follow-up has been determined by the criteria which were defined in previous investigations of chronic heart failure (15, 16). Deaths are classified into 4 kinds: (1) Sudden cardiac death on condition of happening in unconscious condition or within the 1hour after starting any signs or symptoms (2) Progressive heart failure death if it occurs after worsening in the hemodynamic or clinical status (3) Other cardiovascular death refers to the cause which cannot be categorized as sudden cardiac or progressive heart failure death but is associated with cardiac system (4) Non-cardiovascular death. The importance of such classification is to specify the secondary endpoint since former research has declared that sudden cardiac death is the only type of long-term mortality which is related to QT parameters (7). Therefore, sudden cardiac death has been considered the secondary endpoint. The two physicians who were uninformed about patients' ECGs distinguished the type of death by reviewing the hospital documents.

**Statistical Analysis: **SPSS V.23.0 software package was utilized to perform the analysis. Categorical data were portrayed by percentage; mean ± SD was used for continuous data. Based on the normality of continuous variable's distribution, Mann–Whitney U test or Student’s t-test were applied to compare the 

statistical significance of the difference between groups of study. Comparisons between binary parameters were made via chi-square. Kaplan–Meier diagrams were drawn to compare the groups' survival based on QT parameters. For this purpose, continuous ECG parameters were transformed into dichotomized variables. As previously defined, QTc and QTd more than 440ms and 80ms, respectively, were considered abnormally prolonged.

The relationship between arrhythmic events and mortality during hospitalization with their indicators was examined by logistic models and illustrated by odds ratios, their 95% confidence interval (CI), and p-values. Parameters related to long-term mortality were established by Cox’s proportional hazards model and reported by hazard ratios, their CI, and p-values. In multivariate analysis to obtain the best model for mortality prediction, backward stepwise (likelihood ratio) and enter methods were utilized. A p-value cut-off of 0.05 to consider the result of the analysis as significant, has been assumed.

**Figure 1:**
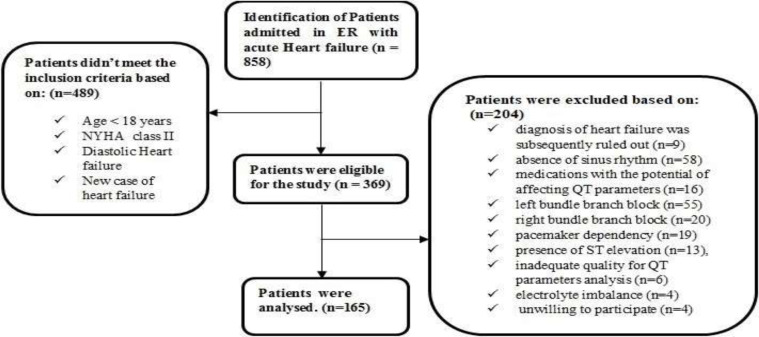
Flowchart of patient selection. Among the 858 patients who were admitted to the emergency department with the primary diagnosis of acute heart failure, 369 were eligible for the study. Two hundred four patients were excluded. Thus, data of 165 patients were analyzed

## Results

The process of choosing the patients is presented in figure 1. Within the time of the study, 858 cases were registered to the emergency ward with the impression of acute heart failure. Among them, 369 had a previous history of heart failure, NYHA Class III-IV, and systolic dysfunction (EF less than 40%); thus, they were eligible for the study. Two hundred four patients were excluded after the primary assessment due to the following reasons: having any final diagnosis except DHF (n=9), absence of sinus rhythm (n=58), using medications with the potential of affecting QT parameters (n=16), left and right bundle branch block (n=75), pacemaker (n=19), presence of ST-elevation (n=13), inadequate quality for QT parameters analysis (n=6), electrolyte imbalance (n=4), and unwilling to participate (n=4). Thus, data of 165 patients were analyzed.

**Demographic and clinical data**

Baseline features of the patients are displayed in table 1. 

**Table 1:** Baseline characteristics of patients

Patient characteristics	Patients ( % or mean± SD)
Age at admission (years)	65 ± 14.1
Male/Female	58/42 %
Current smoker	32.1 %
Substance user	32.7 %
Body mass index (kg/m2)	25.1 ± 4.7
NYHA Classification	
NYHA III	50.3%
NYHA IV	49.7%
Ejection fraction (%)	23.8 ± 8.5 %
**Symptoms**	
Fatigue	12.1 %
Dyspnea	97.6 %
Cough	19.4 %
Dyspnea on exertion	87.3 %
Orthopnea	55.2 %
Paroxysmal nocturnal dyspnea	15.2 %
Nocturia	4.2 %
Weight loss	3.6 %
Weight gain	6.1 %
Peripheral edema	46.1 %
Loss of appetite	17.1 %
Cheyne-stokes respiration	3 %
**Signs**	
Tachycardia(>100/min)	23 %
Thready pulse	6.7 %
Tacypnea(>20/min)	9.1 %
Cooled or mottled extremities	12.1 %
Elevated JVP	14.5 %
Dullness or diminished breath sound in one or both lungs	52.7 %
Rales/Wheeze sound	71.5 %
Third or fourth heart sound	43.6 %
Tricuspid or mitral regurgitation murmur	53.9 %
Hepatomegaly	3 %
Ascites	13.9 %
Anasarca	9.1 %
**Vital status **	
Systolic blood pressure (mm Hg)	129 ± 27
Diastolic blood pressure (mm Hg)	80 ± 15
Heart rate (per minute)	90 ± 18
Temperature °C	36.7 ± 0.45
Oxygen saturation (%)	92.6 ± 6.3
**Medical history**	
Diabetes mellitus	44.2 %
Controlled	36.6 %
Uncontrolled	63.4 %
Hypertension	63.6 %
Controlled	53.3 %
Uncontrolled	46.7 %
Hyperlipidemia	61.8 %
Anemia	60 %
Thalassemia major	2.4 %
Hyperthyroidism	2.4 %
Hypothyroidism	7.3 %
Severe renal impairment	19.4 %
Cerebrovascular disease	8.5 %
Liver disease	3.6 %
Obstructive pulmonary disease (Asthma/COPD)	9.7 %
Previous coronary artery disease	69.1 %
Coronary revascularization (PCI or CABG)	57.9 %
**Etiology of heart failure**	
Hypertension	14.6 %
Valvular heart disease	7.2 %
Ischemic heart disease	69.1 %
Dilated cardiomyopathy	9.1 %
**Baseline ECG characteristics**	
Heart rate (bpm)	90 ± 18
QRS duration (ms)	101 ± 11
QT interval duration (ms)	368 ± 34
QTc interval duration (ms)	440 ± 31.3
QT dispersion (ms)	46 ± 21.6
**Heart monitoring or followed-up ECGs**	
Arrhythmic events	33.9 %
Ventricular premature contraction	28.4 %
Ventricular tachycardia	7.8 %
Ventricular fibrillation	5.4 %
**Radiologic findings**	
Pleural effusion	80 %
Heart enlargement	62.5 %
Calcified plaque (in the aortic arch or descending aorta)	24.1 %
Pulmonary edema	13.8 %
Pulmonary nodular infiltration	3.4 %
Pulmonary patchy infiltration	52.4 %
**Laboratory tests**	
Troponin I (Mic gr/L)	0.14 ± 0.1
White cell blood count (10^3/µL)	8.3 ± 3.4
Hemoglobin (g/dL)	12 ± 2.2
Platelet count (10^3/µL)	201 ± 75
Prothrombin time (sec)	15.7 ± 5.6
Partial thrombin time (sec)	36.7 ± 12
INR (Index)	1.6 ± 1.8
Random blood sugar (mg/dL)	149 ± 80
Sodium (mEq/dL)	137 ± 4
Potassium (mEq/dL)	4.3 ± 0.5
Blood urea nitrogen (mg/dL)	30 ± 18
Creatinine (mg/dL)	1.5 ± 1
SGOT (mg/dL)	108 ± 507
SGPT (IU/L)	99 ± 460
Alkaline phosphatase (mg/dL)	252 ± 130
Albumin (mg/dL)	3.8 ± 0.45
Globulin (g/dL)	2.6 ± 0.6
Total protein (g/dL)	6.3 ± 0.74
Total bilirubin (mg/dL)	1.5 ± 1.5
Direct bilirubin (mg/dL)	0.62 ± 0.83
Triglyceride (mg/dL)	118 ± 59
Cholesterol (mg/dL)	142 ± 44
HDL-CH (mg/dL)	38 ± 14.8
LDL-C (mg/dL)	91 ± 77.5
Uric acid (mg/dL)	8.5 ± 2.9
CK-MB (IU/L)	22 ± 24

NYHA, New York Heart Association classification; JVP, Jugular venous pulse; COPD, Chronic obstructive pulmonary disease; PCI, Percutaneous coronary intervention; CABG, Coronary artery bypass grafting; INR, International normalized ratio; SGOT, Serum glutamic-oxaloacetic transaminase; SGPT, Serum glutamic-pyruvic transaminase; HDL, High-density lipoprotein; LDL, Low-density lipoprotein; CK-MB, creatine kinase-myoglobin binding. Binary variables are expressed by percentage; continuous variables are illustrated as mean ± standard deviation

The population included 96 males and 69 females with an average age of 65 ± 14.1 years. In 53 patients, current smoking was noted (32.1%); 54 patients announced substance consumption which was most opium (32.7%). The mean EF showed severe systolic dysfunction (23.8%). Most of the patients fell within the overweight range according to BMI (25.1±4.7) and were evenly distributed between NYHA classification III and IV (50.3% vs. 49.7%). Dyspnea, orthopnea, and edema were the most common symptoms which occurred during the decompensation (97.6%, 55.2%, and 46.1%). Respiratory sounds (rales/wheeze 71.5%, diminished breath sound 52.7%), heart sounds (third or fourth heart sound 43.6%, tricuspid or mitral regurgitation murmur 53.9%), and extremities edema (46.1%) should be considered as the most common findings in physical examination. Baseline ECG characteristics, laboratory tests, and radiological findings are shown in table1. Diabetes mellitus 44.2% (63.4% of them had uncontrolled HBA1C level according to 2019 ESC guideline), hypertension 63.6% (46.7% had uncontrolled blood pressure according to 2019 ESC guideline), hyperlipidemia 61.8%, previous history of coronary artery disease 69.1% (57.9% underwent (PCI or CABG)) were the most remarkable comorbidities in them. Heart failure was determined based on 4 etiologies in patients: hypertension (14.6%), valvular disease (7.2%), ischemic heart disease (69.1%), and idiopathic dilated cardiomyopathy (9.1%). Table 2 shows medications that have been used before admission and also during hospitalization. Anti-platelet, anti-coagulant, ß-blocking agent, loop diuretic, potassium-sparing diuretic, and statin were the most reported medications before admission and during hospitalization. Table 3 exposes the patient’s outcome. Univariate and multivariate analyses were utilized to distinguish the parameters which can predict arrhythmic events, in-hospital, and long-term mortality.

**Table 2:** Medications that have been used before admission and during hospitalization

Medication	Before admission(%)	Hospital course(%)
Anti-platelet	66.7%	79.4%
Anti-coagulant	30.9%	67.9%
ACE inhibitor	21.8%	37.6%
ARB	35.8%	30.3%
Calcium channel blocker	12.1%	12.7%
ß-Blocking agent	64.8%	83%
Loop diuretics	58.8%	93.9%
Thiazide diuretics	3%	4.2%
Potassium-sparing diuretics	41.8%	56.4%
Statins	52.7%	72.7%
Fibrates	2.4%	0%
Oral antidiabetic drugs	17.6%	12.1%
Insulin	12.1%	17.6%
Digitalis	17.6%	30.9%
Nitrates	43%	64.8%
Allopurinol	3%	6.1%
Catecholamine	0%	7.3%

ACE, Angiotensin-converting enzyme; ARB, Angiotensin receptor blocker.

**Table 3:** Descriptive statistics of patients’ outcome follow-up

Outcome	Patients(%)
Hospitalization course (days)	5 ± 3
Survivors	83%
In-hospital mortality	7.3%
All-cause long-term mortality	9.7%
Sudden death	37.5%
Progression of heart failure	37.5%
Non-cardiac death	25%
Other cardiac cause	0%
Times of Follow up (days)	181 ± 56

The outcome of the patients are presented as (%) or mean±SD of patients

**Arrhythmic events: **Analysis of ECGs indicated that 33.9% (n=56) of DHF patients presented arrhythmic events during the hospitalization. Premature ventricular contraction was illustrated in 28.4% (n=47) of patients’ ECGs; 7.8% (n=13) had ventricular tachycardia and 5.4% (n=9) experienced ventricular fibrillation. DHF patients with and without arrhythmic events had significant differences (p<0.05) regarding the thready pulse (P=0.008), cooled or mottled extremity (P=0.034), hepatomegaly (P=0.004), ascites (P=0.034), anasarca (p<0.001), past drug history of insulin (P=0.040), ß-blocking agent therapy in hospital course (P=0.042), heartbeat (P=0.006), QRS duration (P=0.013), QTc interval (p<0.001), QTd (p<0.001), EF (P=0.004), prothrombin time (P=0.034), and uric acid (P=0.002) in analysis. Table 4 shows the parameters that are associated with the arrhythmic events. Multivariate analysis with backward (likelihood ratio) stepwise selection included all variables which reached statistical significance in univariate analysis. The uric acid level was the limiting factor for these models, which was requested for 38 patients (23%). Thus, we calculated 2 multivariate models with and without uric acid, and as the result of both models, in contrast to other variables which did not remain as an independent predictor in this model, QTc interval (OR 1.085, 95% CI 1.023_1.151, P=0.007) and QTd (OR 1.077, 95% CI 1.020_1.137, P=0.007) were related to arrhythmic events (table 5).

**In-hospital mortality: **The average time to discharge the patients was 5 days (min=1 day, max=18 days). Of 165 patients, 12 (7.3%) died during hospitalization. One patient died due to a non-cardiac cause, while the cause of the others’ death (n=11) was related to cardiac failure. Analysis of parameters that are related to in-hospital mortality is displayed in table 4, including: Extremity edema (P=0.014), thready pulse (P=0.027), elevated JVP (P=0.011), ascites (P=0.049), anasarca (P=0.010), past history of anemia (P=0.049), renal impairment (P=0.001), thiazide (P=0.018), insulin (P=0.026) and allopurinol (P=0.021) therapy at hospitalization, QTc interval (p<0.001), QTd (p<0.001), hemoglobin level (P=0.007), prothrombin time (P=0.047), sodium (P=0.015), potassium (P=0.048), BUN (P=0.013), creatinine (P=0.007), SGOT (P=0.003), SGPT (P=0.049), cholesterol (P=0.013), HDL (P=0.028), and Uric acid (P=0.037). Of these parameters which were entered in the model, only QTc interval (OR 1.116, 95% CI 1.028_1.211), QTd (OR 1.067, 95% CI 1.015_1.123), and history of anemia (OR 1.551, 95% CI 1.373_1.776) showed statistical significance in multivariate analysis; they are demonstrated in table 5. Before backward (likelihood ratio) stepwise selection limiting factors including uric acid, SGOT, SGPT, HDL, and cholesterol were excluded due to their low availability.

**Table 4:** Statistically significant univariate predictors of arrhythmic events and in-hospital mortality (mean±SD or the percentage of the population)

**Univariate analysis of **	**Arrhythmic events**			**In-hospital mortality**		
**Significant predictors**	**Characteristic** **Present**	**Characteristic Absent**	***P***	**Characteristic** **Present**	**Characteristic Absent**	***P***
Peripheral edema ^a^	53.6%	42.2%	0.165	81.8%	43.5%	0.014
Thready pulse ^a^	14.3%	2.8%	0.008	27.3%	5.2%	0.027
Elevated JVP ^a^	14.3%	14.7%	0.946	45.5%	12.3%	0.011
Cooled or mottled extremity ^a^	19.6%	8.3%	0.034	27.3%	11%	0.111
Hepatomegaly ^a^	8.9%	0%	0.004	9.1%	2.6%	0.295
Ascites ^a^	26.8%	7.3%	0.034	36.4%	12.3%	0.049
Anasarca ^a^	21.4%	2.8%	P<0.001	36.4%	7.1%	0.010
Past history of anemia ^a^	63.2%	58.3%	0.547	90.9%	57.8%	0.049
Past history of renal disease ^a^	26.3%	15.7%	0.102	63.6%	16.2%	0.001
Past drug history of insulin ^a^	19.3%	8.3%	0.040	27.3%	11%	0.111
ß-Blocking agent treatment ^a^	91.2%	78.8%	0.042	81.8%	83.1%	0.912
Thiazide diuretics treatment ^a^	5.3%	3.7%	0.694	18.12%	3.2%	0.018
Insulin treatment ^a^	19.3%	16.7%	0.673	45.5%	15.6%	0.026
Allopurinol treatment^ a^	10.5%	3.7%	0.081	27.3%	4.5%	0.021
Heart rate (bpm) ^b^	91.77 ± 20.9	87.06 ± 15.48	0.006	92.55 ± 23.01	90.4± 17.8	0.852
QRS duration (sec) ^b^	104.3 ± 9.21	99.4 ± 11.6	0.013	104.8 ± 8.2	100.8 ± 11.2	0.338
QTc interval (ms) ^b^	465.3 ± 25.8	424.9 ± 19.3	P<0.001	490 ± 14.9	431.1 ± 26.2	P<0.001
QT dispersion (ms) ^b^	66.67 ± 23.6	36.5 ± 13.09	P<0.001	86.36 ± 14.8	44.16 ± 20.2	P<0.001
Ejection fraction (%) ^b^	21.23 ± 8.82	25.28 ± 7.95	0.004	19.09 ± 8.3	24.22 ± 8.3	0.064
Hemoglobin (g/dL) ^b^	11.7 ± 2.67	12.15 ± 1.91	0.329	10.2 ± 2.1	12.1 ± 2.1	0.009
Prothrombin time (sec) ^b^	17.1 ± 8.2	14.9 ± 3.34	0.034	18.2 ± 5.7	15.5 ± 5.5	0.047
Sodium (mg/dL) ^b^	137 ± 5.6	138.4 ± 4.02	0.257	133.3 ± 8.7	138.2 ± 4.1	0.015
Potassium (mg/dL) ^b^	4.39 ± 0.53	4.28 ± 0.54	0.279	4.82 ± 0.86	4.28 ± 0.49	0.048
BUN (mg/dL) ^b^	34.58 ± 21.05	28.14 ± 16.58	0.57	48.8 ± 26.1	29.05 ± 17.1	0.013
Creatinine (mg/dL) ^b^	1.5 ± 0.82	1.51 ± 1.09	0.481	1.48 ± 1.01	1.91 ± 0.81	0.007
SGOT (mg/dL) ^b^	276.6 ± 900.5	31.74 ± 30.5	0.063	1004 ± 1763	32.3 ± 29.7	0.003
SGPT (mg/dL) ^b^	245 ± 819	32.8 ± 29.6	0.144	870 ± 1630	33.8 ± 28.1	0.049
Cholesterol (mg/dL) ^b^	130.8 ± 34.7	150.2 ± 48	0.063	104 ± 43.4	146 ± 43	0.013
HDL (mg/dL) ^b^	33 ± 7.8	40.4 ± 17	0.083	38 ± 14.8	104 ± 43.4	0.028
Uric acid (U/L) ^b^	10.6 ± 3.5	7.53 ± 2.06	0.002	14.1 ± 3.6	7.84 ± 2.04	0.037

All statistically significant p-values (p < 0.05) are in bold. For abbreviations see Table 1.

^a^ Statistical significance of the chi-square test. ^b^ Statistical significance of Man Whitney u test (only for uric acid, student’s t-test was used)

^b^ Mean rank was compared between patients both with and without given characteristics in Man Whitney u test, nevertheless arrhythmic mean was applied for report

**Table 5:** Multivariate analysis of significant predictors of arrhythmic events and in-hospital mortality

**Significant predictors**	**Arrhythmic events**		**In-hospital mortality**	
	**OR (95 % CI) ** ^2^	***P*** ^ 1^	**OR (95 % CI)** ^2^	***P *** ^1^
QTc interval (ms)	1.085 ( 1.023 ; 1.151 )	0.007	1.116 ( 1.028 ; 1.211)	0.009
QT dispersion (ms)	1.077 ( 1.020 ; 1.137 )	0.007	1.067 ( 1.015 ; 1.123 )	0.011
Past history of anemia	1.320 ( 1.057 ; 1.675 )	0.635	1.551 ( 1.373 ; 1.776 )	0.036

All statistically significant p-values (p<0.05) are in bold

^1^ Statistical significance of odds ratio

^2^ Odds ratio calculated by multivariate logistic regression for arrhythmia and in-hospital mortality and its 95 % confidence interval

**Long-term mortality: **Of the 153 patients who were discharged and followed up for an average of 181±56 days, 16(9.7% of all DHF patients) died and 137(83%) subjects survived. At the time of follow-up, no cases did not need to implant a defibrillator or cardiac transplantation. Direct contact with the patients’ families or their hospital documents determined the mode of the death: non-cardiac deaths in 4(25%) patients, progressive heart failure deaths in 6(37.5%) patients, sudden deaths in 6 (37.5%) patients, and other cardiac death in none of them.

**All-cause mortality: **Smoking (P=0.045), weight gain (P=0.037), anasarca (P=0.038), diabetes mellitus (P=0.016), hyperthyroidism (P=0.004), QTc interval (P=0.005), and random blood sugar (P=0.034) were proved as significant predictors in univariate analysis of cox proportional hazards regression (table 6). Among them, only anasarca and random blood sugar lost their statistical significance when they were analyzed by the multivariate method of the Cox proportional hazard model (table 6). 

**Figure 2:**
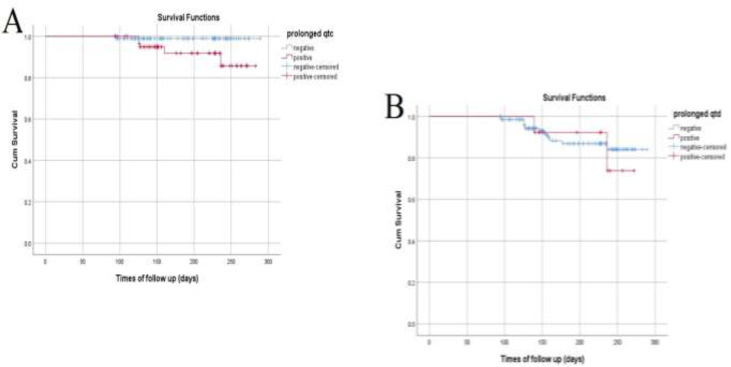
Kaplan-Meier curves for survival compartment

Although QTc interval was related to all-cause mortality in both univariate and multivariate analysis (P=0.005 and p<0.001, respectively), attempts to show a significant association between QTd and all-cause mortality failed. The Survival comparison between the groups with normal and prolonged QT parameters by Kaplan-Meier curves verified our results and is shown in figure 2.

Figure 2a. shows a higher survival rate in the normal QTc interval group rather than the prolonged QTc interval group.

Figure 2b. illustrates no significant difference in survival rate between prolonged and normal QTd.

**Sudden death: **The significant association between age (P=0.034), anasarca (P=0.023), hypertension (P=0.050), hyperthyroidism (P=0.029), and QTc interval (p<0.001) with sudden death was determined by the univariate method of the Cox proportional hazard model (table 6). 

Age and QTc interval preserved their value as significant predictors in multivariate analysis (P=0.010 and P=0.002, respectively). Similar to all-cause mortality, QTc opposed to QTd has presented itself as an independent predictor of sudden cardiac death (table 6). Lots of factors can prolong QT parameters. Although omitting all of them is not possible for the reason of prevention of decreasing the study population, however the most important of them have been listed as exclusion criteria. Valvular heart disease, renal impairment, and digitalis therapy may have an impact on QT parameters; thus, after ignoring the patients with mentioned parameters, analyses were performed again. No change in previous results was observed.

**Table 6:** Univariate and multivariate analysis of significant predictors of all-cause and sudden death mortality

**Univariate analysis of**	**Long-term mortality**			
**Significant predictors**	**All-cause **		**Sudden death**	
	**HR (95 % CI)** ^ 2^	***P *** ^1^	**HR (95 % CI) ** ^2^	***P *** ^1^
Age	1.011 ( 0.974 ; 1.050 )	0.567	1.092 ( 1.006 ; 1.185 )	0.034
Smoking	0.363 ( 0.135 ; 0.976 )	0.045	0.241 ( 0.044 ; 1.316 )	0.100
Weight gain	0.262 ( 0.074 ; 0.925 )	0.037	0.318 ( 0.037 ; 2.738 )	0.297
Anasarca	0.261 ( 0.073 ; 0.931 )	0.038	0.134 ( 0.024 ; 0.759 )	0.023
Diabetes mellitus	0.271 ( 0.094 ; 0.785 )	0.016	1.237 ( 0.225 ; 6.817 )	0.807
Hypertension	1.731 ( 0.649 ; 3.013 )	0.273	1.291 ( 1.046 ; 1.593 )	0.050
Hyperthyroidism	0.110 ( 0.025 ; 0.493 )	0.004	0.088 ( 0.010 ; 0.776 )	0.029
QRS duration (ms)	1.011 ( 0.967 ; 1.058 )	0.622	1.015 ( 0.942 ; 1.094 )	0.696
QTc interval (ms)	1.018 ( 1.005 ; 1.031 )	0.005	1.032 ( 1.014 ; 1.050 )	P<0.001
QT dispersion (ms)	1.002 ( 0.980 ; 1.022 )	0.891	1.015 ( 0.989 ; 1.042 )	0.253
Random blood sugar	1.004 ( 1.000 ; 1.009 )	0.034	0.995 ( 0.982 ; 1.009 )	0.482
Multivariate analysis of	All-cause		Sudden death	
Significant predictors	HR (95 % CI) ^2^	*P * ^1^	HR (95 % CI) ^2^	*P * ^1^
Age	1.029 ( 0.019 ; 1.076 )	0.215	1.106 ( 1.025 ; 1.194 )	0.010
Smoking	0.280 ( 0.091 ; 0.858 )	0.026	0.101 ( 0.014 ; 0.744 )	0.124
Weight gain	0.116 ( 0.019 ; 0.705 )	0.019	0.442 ( 0.328 ; 0.597 )	0.690
Anasarca	0.282 ( 0.053 ; 1.493 )	0.137	0.132 ( 0.014 ; 1.232 )	0.076
Diabetes mellitus	0.212 ( 0.053 ; 0.856 )	0.029	0.771 ( 0.605 ; 0.981 )	0.067
Hypertension	1.187 ( 0.533 ; 1.841 )	0.311	1.347 ( 1.043 ; 1.671 )	0.550
Hyperthyroidism	0.019 ( 0.002 ; 0.165 )	P<0.001	0.162 ( 0.003 ; 0.497 )	0.385
QRS duration (ms)	1.014 ( 0.954 ; 1.077 )	0.659	0.926 ( 0.791 ; 1.084 )	0.340
QTc interval (ms)	1.041 ( 1.015 ; 1.067 )	0.002	1.063 ( 1.023 ; 1.105 )	0.002
QT dispersion (ms)	1.012 ( 0.990 ; 1.034 )	0.782	1.018 ( 0.941 ; 1.055 )	0.192
Random blood sugar	1.005 ( 0.999 ; 1.012 )	0.119	0.997 ( 0.970 ; 1.026 )	0.846

All statistically significant p-values (p<0.05) are in bold ^1^ Statistical significance of hazard ratio ^2^ Hazard ratio calculated by multivariate Cox proportional hazard regression for long-term mortality and its 95 % confidence interval

## Discussion

Our outcomes illustrate that adverse events can be predicted with QTc interval and QTd as ventricular repolarization indicators. The unique point of our research is the comprehensive evaluation of repolarization parameters besides the other characteristics which were carefully characterized, in a way that resembles risk stratification design.

Mortality due to inadequate cardiac output can be determined by a variety of factors. Systolic or diastolic cardiac dysfunction and valvular or vascular pathology were used to be the most noticeable factors, but recent efforts have been dedicated to investigating the electrophysiological alterations such as cardiac arrhythmias. The cardiac cycle consists of ventricular depolarization and repolarization, and arrhythmia can occur in both. Ventricular activation or depolarization is represented by QRS complex. Prolonged QRS complex was assumed as ventricular activation dys-synchrony and plays the main role in the prediction of arrhythmic events and mortality (5, 17-21). In contrast to previous studies, we focused to explore the association between ventricular repolarization and prognostic factors, including arrhythmic events, in-hospital, and long-term mortality; hence, the patients with bundle branch block were excluded and those who had normal QRS (<120 ms) duration was entered. According to this approach, none of the multivariate models suggest QRS duration as a predictor of arrhythmic events, in-hospital, and long-term mortality. Ventricular relaxation or repolarization alterations predispose to lethal arrhythmias in a sequence of events. The variety of heart diseases causes structural and electrical modifications in the ion channels of the myocytes, leading to changes in the myocytes’ action potentials, including their refractory period and conduction velocity, which results in heterogeneity and fluctuations in repolarization, promoting lethal arrhythmia (22, 23). Morphological remodeling of the histological substrate (myocyte hypertrophy, disarray, fibrosis, etc.), especially ion channel (down-regulation of potassium channels; on the contrary, inactivation of sodium channels, and the release and storage of calcium in the sarcoplasmic reticulum) were found to be the pathological basis of ventricular repolarization inhomogeneity (24). Thus, ventricular repolarization measurement is recommended to stratify arrhythmic events; we analyzed QTc interval duration and QTd for this purpose.

Prolonged QTc interval has been offered as a powerful predictor for ventricular arrhythmia, in-hospital, and long-term mortality in heart failure patients (6, 25, 26). Temizkan et al. (11) represented a hypothesis indicating that monitoring the QTc interval act as an effective instrument in evaluating the DHF patients in the emergency ward which guides us to make a decision about a patient’s discharge or transfer to cardiac care unit. Vaclavik et al. (17) declared that prolonged QTc interval is related to in-hospital mortality, as opposed to long-term mortality. 

In some reports, a lack of correlation between QTc interval with arrhythmia, in-hospital mortality, and long-term mortality (7, 17) was found in acute and chronic heart failure patients subsequently. Breidthardt et al. (5) in a study on 173 acute established heart failure patients revealed a negative association between the QTc interval and long-term mortality. Overall, outcomes on the predictive value of QTc interval in heart failure are controversial. In the same line with previous studies, we were able to indicate a significant relationship between QTc interval as a predictor of arrhythmic events, in-hospital and long-term mortality.

It seems that sole assessment of QT interval to have an arrhythmic risk appraisal is not enough. QTd as a tool that accurately demonstrates unequal action potentials’ prolongation, incongruity of the duration of the refractory periods, and the conduction velocities of adjacent myocardial regions, is more advantageous to represent ventricular repolarization disturbance (3). The prognostic utility of QTd about arrhythmic events, in-hospital and long-term mortality in heart failure patients has been confirmed by some projects (4, 9, 27, 28). Padmanabhan et al. (26) determined QT interval dispersion as a predictive parameter of all-cause mortality from a cohort of 2265 patients with reduced EF. Nevertheless, some recent papers have failed to demonstrate the predictive role of QTd for arrhythmic events, in-hospital and long-term mortality in heart failure patients (7, 8). Brendorp et al. (29) rejected the predicting role of QTd regarding the all-cause mortality after inspecting the 703 heart failure patients with reduced EF. In our project, effort to corroborate a significant association between QTd with arrhythmic events and in-hospital mortality was fruitful. In contrast, using Cox regression to predict long-term mortality (all-cause and sudden death) for QTd failed.

These findings have important clinical benefits. Risk stratification of DHF cases is applicable by these ECG parameters because of their mortality anticipating features; also based on ECG's unique features (the most accessible, frugal, easy to work with, quick responding, repeatable, and objective), it can be more practicable compared to other prognostic tools. Rapid intervention by pharmacological (e.g.levosimendan) or invasional therapeutic approach in high-risk patients recognized by these parameters may be so beneficial (5). Despite its prognostic utility, ECG helps to diagnose DHF, especially in identifying the etiology of decompensation containing arrhythmic or ischemic events, so the guidelines of AHA or ESC recommended the ECG as a primary workup for the management of DHF patients.

Although the research has reached its aim, some restrictions exist. At first, despite the remarkable patients’ referral to our hospital (n=858), stringent criteria made our sample size small (n=165, 19.2%), so similar studies with more cases are recommended. Second, baseline computer-derived ECG contained all the parameters except QTd; however, QTd was calculated manually by a single expert cardiologist who was uninformed about clinical details and outcomes. Although all efforts were made, the manual calculation may affect results, particularly long-term mortality. However, based on the report by Glancy et al. (30) difference between values of QTd measured by manual and automatic methods is scarce, and errors in manual calculating do not impress the outcomes. Third, uric acid, SGOT, SGPT, HDL, and cholesterol were limiting parameters and were kept out of the multivariate logistic regression model; thus, their impact on QT parameters outcome predictability was not elucidated.

Our research had three specific strengths. First, defining 13 inclusion and exclusion criteria to achieve the purest association between QT’s parameters and the outcome made our research more accurate compared to similar works. Second, accompanying factors, including demographic data, signs and symptoms, physical examination findings, comorbidities, past social and medication history, hospital medication, etiology of heart failure, laboratory and radiologic parameters neglected in previous investigations, were analyzed in combination with ECG parameters simultaneously. Third, considering the outcome based on the occurrence of arrhythmic events, in-hospital and long-term mortality is more reliable than separately evaluating them.

The present prospective study on decompensated heart failure patients suggests considering QTc interval as a prognosticator of arrhythmic events, in-hospital mortality, and sudden death in the long term. Meanwhile, QT interval dispersion is the determinant factor of arrhythmic events and in-hospital mortality. According to their utility, they should be measured for risk stratification of ventricular repolarization arrhythmia and death in DHF patients in daily clinical practice.
